# A high‐quality walnut genome assembly reveals extensive gene expression divergences after whole‐genome duplication

**DOI:** 10.1111/pbi.13350

**Published:** 2020-02-24

**Authors:** Junpei Zhang, Wenting Zhang, Feiyang Ji, Jie Qiu, Xiaobo Song, Dechao Bu, Gang Pan, Qingguo Ma, Jiaxin Chen, Ruimin Huang, Yingying Chang, Dong Pei

**Affiliations:** ^1^ State Key Laboratory of Tree Genetics and Breeding Key Laboratory of Tree Breeding and Cultivation of the State Forestry and Grassland Administration Research Institute of Forestry Chinese Academy of Forestry Beijing China; ^2^ Shanghai Key Laboratory of Plant Molecular Sciences College of Life Sciences Shanghai Normal University Shanghai China; ^3^ Key Laboratory of Intelligent Information Processing Advanced Computer Research Center Institute of Computing Technology Chinese Academy of Sciences Beijing China; ^4^ Tibet Agriculture and Animal Husbandry University Linzhi, Tibet China

**Keywords:** genome, Juglans, WGD

The common walnut *Juglans regia* and its related species in the genus *Juglans* are important economic trees, which have been widely grown for nut and wood productions in many counties. In the *Juglans* genus, there are ~ 21 diploid species all with 2*n* = 32 chromosomes, which can be divided into four major sections based on phylogenetic analyses and fruit morphology – *Cardiocaryon* (e.g. *J. cathayensis* and *J. mandshurica*), *Juglans* (e.g. *J. sigillata* and the most popular one *J. regia*), *Trachycaryon* (e.g. *J. cinerea*) and *Rhysocaryon* (e.g. *J. hindsii*, *J. nigra* and *J. microcarpa*) (Aradhya *et al.*, 2007). For walnuts, the draft genome sequences of several *Juglans* species had been generated from second‐generation sequencing platform (*J. regia Chandler* N50: 465Kb, Martınez‐Garcıa *et al.*, 2016; *J. regia Chandler* N50: 640Kb, Stevens *et al.*, 2018). Very recently, the *J. microcarpa* × *J. regia* hybrid was sequenced using single molecule sequencing technology, and the new methods for haplotype phasing facilitated the genome assembling of the two species (*J. regia Serr* N50: 2.90 Mb, Zhu *et al.*, 2019).

In this study, we aimed to generate a high‐quality reference‐level *de novo* assembly and gene annotations of *J. regia* for genetic and genomic studies in walnut. To search for *J. regia* with low heterozygous rate, we selected 7 diverse *J. regia* accessions for Illumina sequencing to estimate the whole‐genome‐level heterozygosity. The accession Zhongmucha‐1 (an ancient tree from Tibet, China; N29°04.889’ E92°44.432’; 3277 ASL) with the lowest (0.385%) heterozygosity was used to generate high‐quality genome sequences. The resulting genome assemblies of Zhongmucha‐1 contain a total of 353 contigs, with the contig N50 size of 3.34 Mb. Combined with Hi‐C data generated in this work and the linkage and physical maps reported previously (Luo *et al.*, 2015; Zhu *et al.*, 2019), the contigs were anchored and ordered, generating chromosome‐level sequences of 540 Mb. We found ~ 95% of the RNA‐seq reads and 97.25% of the Illumina sequencing reads could be aligned onto the final assembly. The completeness of the genome assembly was then evaluated using BUSCO data sets (Simão *et al.*, 2016), and ~ 94% of the core eukaryotic genes were able to be retrieved. To annotate the walnut genome for protein‐coding genes, we used a hybrid gene prediction protocol with combinations of *ab initio* gene predictions and homologs sequence searching, which was further integrated with RNA‐seq data from 9 representative tissues in *J. regia* (Figure 1a).

**Figure 1:**
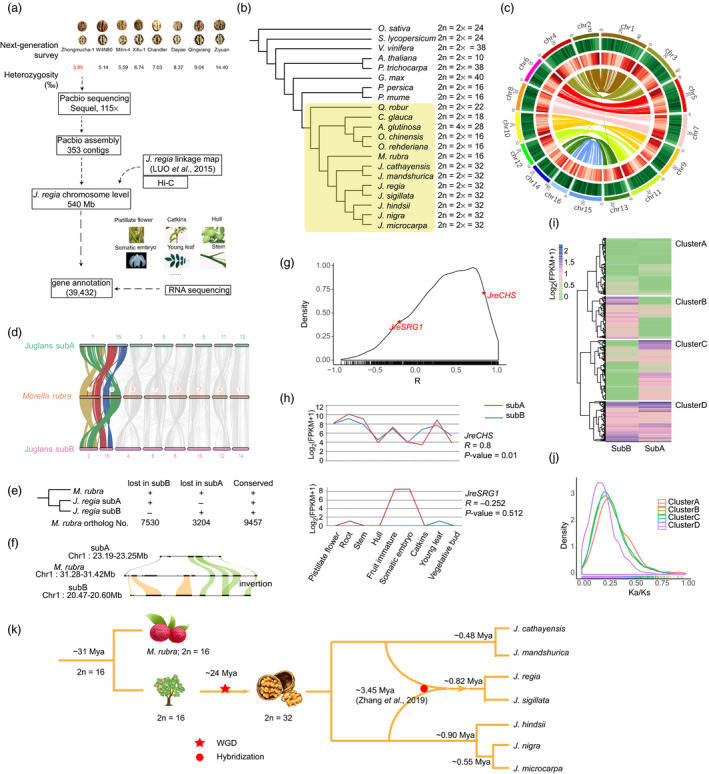
(a) Sequence assembly flow chart for *Juglans regia* ‘Zhongmucha‐1’. (b) Phylogenetic tree of angiosperm species on the basis of orthologs of single‐copy gene families. Yellow rectangle region represents fagales species. (c) Self‐alignment of walnut genomes. The circus map shows, from outside to inside, ideograms of the sixteen chromosomes, density of TE, density of genes and syntenic blocks. (d) Collinear relationship at the chromosome level between *Morella rubra* and *Juglans regia* sub‐genomes. (e) The summary of gene losses in two sub‐genomes of *J. regia* through comparisons with *M. rubra*. ‘+’ and ‘‐’ indicate the gene retention and gene loss, respectively. (f) A local comparison for genes in *Morella rubra* and their orthologs in *J. regia* sub‐genomes. The black boxes indicate genes, the grey arc ligature indicates gene lost in *J. regia* subB, the yellow arc ligatures indicate genes lost in *J. regia* subA, and the green arc ligatures indicate genes conserved in *J. regia* subA and subB. (g) Expression correlation of sub‐genome paired genes. Frequency distributions are shown for Spearman correlation coefficient calculated from expressions of paired genes in nine tissues. Among them, (h) the detailed expression profiles in nine tissues are shown for *JreCHS* and *JreSRG1*. (i) Heatmap and cluster of gene pairs between sub‐genomes based on their expressions in fruit immature. (j) Frequency distributions of Ka/Ks values for four clusters. (k) A summarized model proposed for the phylogeny of *Juglans*. The divergence time of *M. rubra* and *J. regia* was estimated ~ 31 Mya. The walnut genome had undergone a whole‐genome duplication event from 2n = 16 to 2n = 32 chromosomes around 24 million years ago. The rediploidization event was likely to occur soon after WGD for the *Juglans* genus. *J. regia* and its landrace *J. sigillata* arose as a hybrid between the American and the Asian lineages around 3.45 million years ago.

Based on the collinear relationship with our *J. regia* genome, the scaffolds from other five *Juglans* species (*J. cathayensis*, *J. mandshurica*, *J. sigillata*, *J. hindsii* and *J. nigra*) were anchored onto chromosomes followed with ordering and orientation, generating 16 pseudochromosomes for each *Juglans* species. Taken together, coupled with the high‐quality assembly of *J. microcarpa* (Zhu *et al.*, 2019), the chromosome‐level sequences are now available for totally seven species from the three major sections in *Juglans*. Through aligning the assemblies of the six related species with that of *J. regia*, we identified highly confident variants within coding regions, including 2 270 791 single nucleotide polymorphisms (SNPs), 235 764 small indels of ≤ 20 bp and 52 363 structural variants (SVs). The genome assemblies of all *Juglans* and the variation data have been deposited at http://xhhuanglab.cn/data/juglans.html, and the assembly of *Juglans regia* can also be downloaded from BIGD under Bioproject number PRJCA002070.

Using single‐copy genes, we constructed a phylogenetic tree for the seven *Juglans* species, their close relatives with reported genome sequences and the core eudicots genomes, along with *Oryza sativa* from monocots as outgroup (Figure 1b). Self‐alignment of the walnut genome sequences based on the 39 432 gene models identified 11 446 paralogous gene groups with 555 synteny blocks, which indicated that the gene pairs were actually due to one‐to‐one chromosome pairs on the walnut genome (dividing into two sub‐genomes – subA and subB in this work; Figure 1c). A total of 11 938 (between red bayberry and walnut subA) and 7978 (between red bayberry and walnut subB) one‐to‐one orthologous gene pair blocks were found and used to visualize the detailed orthologous chromosome‐to‐chromosome relationships (Figure 1d). There were 9457 cases with both copies retained in *J. regia*, 7530 cases with singletons retained in subA and 3204 retained in subB, meaning more than half of the duplicated gene pairs had lost one copy in *J. regia* after the WGD (Figure 1e). According to the estimation of the WGD time (~24.48 million years ago), coding genes were lost at a rate range from 0.56% to 1.62% per million years for each chromosome.

We further focused on 6981 one‐to‐one collinear gene pairs between sub‐genomes to compare the expression patterns in each pair. The correlation coefficient of the expression levels of the two copies in nine tissues was calculated for each gene pair (Figure 1g), and ~22% pairs showed significant correlations (850 pairs with *P* < 0.05). The other ~78% pairs showed weak or no expression pattern correlations between copies in subA and subB, suggesting the duplicates of each other begin to diverge in expression levels and patterns. As a typical example for co‐expressed gene pairs (Figure 1h), the two copies *JreCHS_subA_* and *JreCHS_subB_* (a key enzyme involved in the biosynthesis of flavonoids) showed closely correlated gene expressions, which may indicate that both copies were responsible for the biosynthesis of the flavonoids with less functional divergence. Another contrasting example is *JreSRG1* (a gene involved in plant senescence; Figure 1h), of which the subA copy were normally expressed in immature fruit and somatic embryo while the subB copy had no transcripts in these tissues, with numerous changes in promoter regions between the two copies (sequence identity = 45%). The abundant gene copies exhibiting diverse expression patterns were probably due to the sequence divergence in the promoter and other regulation regions (e.g. UTRs and enhancers) between sub‐genomes. We then performed hierarchical clustering for these gene pairs based on their expression patterns in each tissue. For the tissue ‘immature fruit’ (Figure 1i), gene pairs could be classified into four distinctive clusters. Frequency distributions of the Ka/Ks ratio in the four clusters showed the clusterD (high expressions in both copies) had the lowest Ka/Ks ratio peak value (ClusterA: 0.2582; ClusterB: 0.2257; ClusterC: 0.2243; ClusterD: 0.1736), which suggested genes in this cluster tended to be conserved (Figure 1j).

Along with *J. microcarpa* (Zhu *et al.*, 2019), now we totally have 7 well‐assembled *Juglans* genomes. Through the estimation of the peak Ks value of whole‐genome orthologous genes between *J. regia* and each of the related species, *J. regia* speciated with *J. sigillata* roughly 0.84 million years ago and with other related species ~2.64 million years ago, much later than the WGD event (24.48 million years). Moreover, based on the Ks values, we can estimate the divergence time between *M. rubra* and *J. regia* was about 31 million years ago close to previous estimation (Jia *et al.*, 2019), and the ancestry of the *J. regia* and *J. sigillata* genome and the origin of *J. regia* and *J. sigillata* was dated to ~0.82 Mya. Recently, Zhang *et al*, 2019 discovered that *J. regia* (and its landrace *J. sigillata*) arose as a hybrid between the American and the Asian lineages around 3.45 million year ago. Based on these clues, now we proposed a summarized model for the phylogeny of *Juglans*, as displayed in Figure 1k.

## Author contributions

D.P. conceived and designed the experiments. J.Z, F.J., X.S., G.P., Q.M. and Y.C performed the experiments. W.Z., F.J., J.Q., D.B., R.H. and J.C. analysed the genome data. W.Z. and D.P. wrote the manuscript.

## Conflict of interest

The authors declare that the research was conducted in the absence of any commercial or financial relationships that could be construed as a potential conflict of interest.
